# Socioeconomic inequalities in access to skilled birth attendance among urban and rural women in low-income and middle-income countries

**DOI:** 10.1136/bmjgh-2018-000898

**Published:** 2018-12-01

**Authors:** Gary Joseph, Inácio Crochemore Mohnsam da Silva, Aluísio J D Barros, Cesar G Victora

**Affiliations:** International Center for Equity in Health Post-Graduate Program in Epidemiology, Federal University of Pelotas, Pelotas, Brazil

**Keywords:** maternal health, birth attendance, low and middle-income countries, socioeconomic inequalities, urbanization, Maternalhealth, birth attendance, low andmiddle-income countries, socioeconomic inequalities, urbanization

## Abstract

**Introduction:**

Rapid urbanisation is one of the greatest challenges for Sustainable Development Goals. We compared socioeconomic inequalities in urban and rural women’s access to skilled birth attendance (SBA) and to assess whether the poorest urban women have an advantage over the poorest rural women.

**Methods:**

The latest available surveys (DemographicHealth Survey, Multiple Indicators Cluster Surveys) of 88 countries since 2010 were analysed. SBA coverage was calculated for 10 subgroups of women according to wealth quintile and urban-rural residence. Poisson regression was used to test interactions between wealth quintile index and urban-rural residence on coverage. The slope index of inequality (SII) and concentration index were calculated for urban and rural women.

**Results:**

37 countries had surveys with at least 25 women in each of the 10 cells. Average rural average coverage was 72.8 % (ranging from 17.2% % in South Sudan to 99.9 % in Jordan) and average urban coverage was 80.0% (from 23.6% in South Sudan to 99.7% in Guyana. In 33 countries, rural coverage was lower than urban coverage; the difference was significant (p<0.05) in 15 countries. The widest urban/rural coverage gap was in the Central African Republic (32.8% points; p<0.001). Most countries showed narrower socioeconomic inequalities in urban than in rural areas. The largest difference was observed in Panama, where the rural SII was 77.1% points larger than the urban SII (p<0.001). In 31 countries, the poorest rural women had lower coverage than the poorest urban women; in 20 countries, these differences were statistically significant (p<0.05).

**Conclusion:**

In most countries studied, urban areas present a double advantage of higher SBA coverage and narrower wealth-related inequalities when compared with rural areas. Studies of the intersectionality of wealth and residence can support policy decisions about which subgroups require special efforts to reach universal coverage.

**Key questions**

**What is already known?**

- Rapid urbanisation in low-income and middle-income countries results in large contingents of urban poor families, in which women and children are exposed to high risks of morbidity and mortality. (Box 1)
- Progress to universal health coverage tends to follow a common pathway with the urban rich being the first to obtain universal coverage and the rural poor, the last to be reached.

**What are the new findings?**

- By studying coverage with skilled birth attendance (SBA), we show that wealth-related inequalities are superimposed on urban-rural inequalities.
- In almost all countries, coverage among rural poor women was lower than for the urban poor, and socioeconomic inequalities were wider in rural than in urban areas.

**What do the new findings imply?**

- Double stratification of health outcomes according to wealth and residence may guide policy decisions on which subgroups require special efforts to reach universal SBA coverage.

## Introduction

In today's increasingly global and interconnected world, the urban population has grown rapidly from 746 million in 1950 to 3.9 billion in 2014.^1^ In 2016, 54% of the world’s population were living in urban settlements, and by 2050, this is expected to reach 60%, as a result of large-scale migration from rural to urban areas, mostly in low-income and middle-income countries (LMICs).^2 3^ Historically, the urbanisation process has been related to important economic and social transformations such as lower fertility, higher level of literacy and education and greater access to health services.^2^ However, far from being good news, rapid and unplanned urban growth in LMICs can be seen as a threat to sustainable development and healthy living, when the necessary infrastructure is not developed or when policies are not implemented to guarantee equitable benefits from life in the cities.^2^ Previous studies have shown that, in less developed countries, high levels of urbanisation are likely to be associated with important inequalities in maternal health services, large impoverished and marginalised settlements and high maternal and newborn mortality among the urban poor.^4–6^

Indeed, some authors suggested that poor urban dwellers may fail to present improved levels of health status and healthcare than poor families residing in rural areas—that is, an ‘urban advantage’ would not exist.^4^ Other authors, however, do not share this view. In a study on facility delivery conducted by Channon *et al*, using data from 33 LMICs, progress to universal health coverage followed a common pathway with the urban rich being the first to obtain universal coverage followed by the rural rich, the urban poor and finally the rural poor, who were the last to be reached.^7^

Because data on urban poor or slum dwellers are usually limited or not available, most studies tended to ignore this group and to report inequalities in health status or access to services based on average differences between urban and rural areas or between rich and poor, without considering both dimensions simultaneously. This usually leads to the conclusion that most countries present better health status and higher coverage in urban than in rural areas, which may not be true for the poor. A study conducted by Matthews *et al*, using nationally representative surveys from 30 LMICs, concluded that there are two main patterns of urban inequality in LMICs: (1) massive exclusion, in which most of the population do not have access to services and (2) urban marginalisation, in which only the poor are excluded. They also proposed that, at country level, these two types of inequality can be further subdivided on the basis of rural access levels.^4^ The studies by Matthews and Channon^4 7^ represent important advances in the literature, but we believe that greater availability of recent surveys may allow further analyses and identification of additional patterns to those described by these authors.

In our analyses, we used the term ‘bottom inequality’ and ‘top inequality’ to refer to the above-mentioned patterns of marginalisation and massive exclusion, respectively.^8^ Bottom inequality refers to a pattern where coverage is markedly lower among the poorest quintile, with all other four quintiles showing relatively high coverage; top inequality is present when the richest quintile shows high coverage while the rest of the population lags behind.

We used the coverage of births attended by skilled health personnel (SBA) to assess inequalities between urban and rural residents, further stratified by wealth quintiles. SBA was chosen because it reveals important socioeconomic inequalities in most countries. In addition, it was one of the key Millennium Development Goals indicators to monitor progress in maternal health until 2015 and remains as one essential indicator for the Sustainable Development Goals (SDGs). The SDG 3.6 goal is aimed at increasing coverage to 90% by 2030.^9–11^

Analyses of intersectionality are gaining importance and visibility in order to identify subgroups of the population that may require stronger efforts in order to reach universal coverage. We aimed to compare socioeconomic inequalities in urban and rural women’s access to delivery care and in particular to assess whether the poorest urban women have an advantage over the poorest rural women.

## Methods

We used data from Demographic Health Surveys (DHS) and Multiple Indicators Cluster Surveys (MICS) that are large and nationally representative surveys that provide information on a wide range of health indicators that are normally comparable across countries. DHS and MICS are based on multistage sampling designs and used standardised questionnaires to collect information from all women living in the sampled households. The surveys are conducted and implemented by the national statistics agencies of each country under the supervision of Unicef (MICS) and USAID (DHS). Ethical approval was the responsibility of the institutions in charge of each survey, ensuring the complete confidentiality of the participants. More details on DHS and MICS can be found elsewhere.^12 13^

We analysed the latest available surveys for 88 countries since 2010 and for which information was available on birth attendance, urban-rural residence and socioeconomic position (SEP) of the household. Of these surveys, 47 were DHS and 41 MICS.

Participants were women in reproductive age from 15 to 49 years old with information on birth attendance in the 3 (DHS) or 2 years (MICS) preceding the survey. For MICS, data on household assets and place of residence were retrieved from the women’s dataset and for DHS from the children’s dataset. These data were then matched to household file that contained information on asset indices.

The dependent variable was skilled birth attendance coverage, defined as whether the delivery took place in the presence of a qualified personnel: a doctor, nurse, midwife, auxiliary midwife or other cadres that each country individually considers as such. This information was collected in the surveys questionnaires through unprompted answers to the question *‘Who assisted with the delivery of (Name of the child*)?’. Such question has been validated in two studies conducted in Mexico and Kenya and demonstrated to be reliable to generate estimates of SBA coverage for use at population level.^14 15^

Place of residence was defined as either urban or rural, based on criteria defined by each county.

The classification of households according to SEP is based on asset indices. Household questionnaires collect information on household appliances (such as televisions, refrigerators and other appliances), characteristics of the building (materials used for the walls, floor and roof and presence of electricity, water supply and sanitary facilities) and other variables related to economic status (ownership of the house, of land or livestock). Initially, these variables are included in a principal component analysis (PCA) for all households in the sample, excluding variables that are only relevant for one domain (eg, livestock or land size which only apply to rural areas). Next, two separate PCAs are carried out for urban and rural households, including all relevant variables in each domain, and the values of these scores are stored. Then, the results of the separate urban and rural PCAs are used to predict the joint PCA scores through linear regression. The intercept and slope from each of the two regressions are used to scale the urban and rural PCA scores for each household into a single, combined score. This is the asset index, which may then be split into quintiles, where the first quintile (Q1) represents the 20% poorest of household in the survey sample and the fifth quintile (Q5) represents the richest 20%.^16^

The descriptive analysis included the estimation of SBA coverage at both national level and for the 10 combinations of wealth quintiles index by urban/rural residence. All surveys without any women in at least one cell of the 10 combinations were excluded from the analysis. Surveys with a fewer than 25 women in at least one cell were partially analysed and are presented in the appendix.

Poisson regression model was used to test the interaction between wealth quintile index and urban/rural residence on SBA coverage. Interactions were highlighted when the p-value was equal or lower than 10%. We used Student’s t-test to calculate the significance level for the difference in coverage between the urban and rural poorest groups. The results are presented graphically using scatter diagrams and equiplots (http://www.equidade.org/equiplot), in which each dot represents coverage in a given wealth quintile, and a horizontal line links the richest and poorest quintiles.

Two summary measures of inequality, which take into account the entire distribution of SBA coverage over the five wealth quintiles, were calculated separately for urban and rural women: the slope index of inequality (SII) and the concentration index (CIX). Their calculation involves a weighting by the size of the samples, which allow to produce a single number that describes inequality among all subgroups.^9 17^ SII measures absolute inequality and represents the absolute difference in per cent points between the fitted coverage levels at the two extremes of the wealth distribution (poorest and richest subgroups). SII takes values from −100 to +100, where zero (0) means absence of inequality. Positive values indicate that the outcome is more prevalent in the rich, while negative values mean that the indicator is more prevalent in the poor.

A similar approach is used to calculate the CIX by ranking individuals according to SEP on the x-axis and plotting the cumulative intervention coverage on the y-axis. The CIX is similar to the Gini index that is commonly used to describe the concentration of income. It also takes values from −100 to 100, where zero means no inequality between subgroups. Negative values indicate concentration of the health indicator among the poorest, and positive values imply the highest concentration among the richest.^9 17^ More details about CIX and SII can be found elsewhere.^9 17^ These two measures were chosen based on that they are the most common summary indices used in the literature to measure health inequalities in subgroups with natural ordering such as wealth quintiles, and because it is important to report on absolute as well as relative inequalities.^9 17^

Countries were grouped according to the seven Unicef regions: Central and Eastern Europe and the Commonwealth of Independent States (CEE & CIS), East Asia and Pacific, Eastern & Southern Africa, Latin American and Caribbean, Middle East & North Africa, South Asia and West & Central Africa.

Sampling weights and the clustered nature of the survey sample were taken into consideration in the analyses. All the analyses were carried out using Stata V.13.1.

## Results

Out of 88 available national surveys, we excluded nine (six DHS, three MICS) in which there were no women in 1 of the 10 cells representing the combination of urban/rural residence and wealth quintiles: Cameroon (2011), Congo (2011), Côte d'Ivoire (2011), Egypt (2014), Haiti (2012), Turkmenistan (2015), Ukraine (2012), Uruguay (2012) and Zimbabwe (2015). Most often, there were no rural women in the wealthiest quintile.

Data on the remaining 79 surveys are available in (online supplementary table 1). Forty-two surveys (18 DHS, 24 MICS) had at least one cell with a denominator lower than 25 women. Our analyses are based on 37 surveys (23 DHS, 14 MICS) with more than 25 women in each cell.

10.1136/bmjgh-2018-000898.supp1 Supplementary data

We focused the presentation of results on the magnitude and direction of differences between urban and rural areas. We also report the significance level of the differences, when applicable.

Taking all countries together (table 1), the national average coverage was 76.4% ranging from 19.4% in South Sudan to 99.6% in Thailand. Rural average coverage was 72.8%, from 17.2% in South Sudan to 99.9% in Jordan, while in urban areas the average was 80.0% with South Sudan having the lowest (23.6%) and Guyana the highest coverage level (99.7%). Tests for the interaction between wealth quintile index and urban/rural residence on SBA coverage showed p levels below 0.05 for 16 of the 37 countries. Another three countries had p levels between 0.05 and 0.10 (online supplementary table 1).

**Table 1*:** Mean and range of the coverage and inequality indicators

	Mean	Lowest	Highest
National coverage	76.4	19.4 (South Sudan)	99.9 (Jordan)
Rural coverage	72.8	17.2 (South Sudan)	99.9 (Jordan)
Urban coverage	80.0	23.6 (South Sudan)	99.7 (Guyana)
Rural absolute inequality (SII)	34.8	0.7 (Jordan)	78.3 (Panama)
Urban absolute inequality (SII)	25.6	−0.9 (Kyrgyzstan)	68.8 (Chad)
Rural relative inequality (CIX)	12.2	0.1 (Jordan)	47.9 (Nigeria)
Urban relative inequality (CIX)	5.9	−0.1 (Belize, Kyrgyzstan)	26.6 (South Sudan)
Urban-rural difference in coverage in the poorest quintile	10.7	−10.6 (Malawi)	34.0 (CAR)
Urban-rural difference in absolute inequality (SII)	−9.2	−77.1 (Panama)	64.2 (Chad)
Urban-rural difference in national coverage	7.2	−1.3 (Malawi)	32.8 (CAR)

CIX, concentration index; SII, slope index of inequality.

In term of SII and CIX, the lowest magnitude of inequality in urban areas was observed in Kyrgyzstan (both for absolute and relative inequalities) and Belize (for relative inequality), while Jordan presented the smallest rural inequalities. The negative values for SII and CIX in Kyrgyzstan and Belize indicate that the poor had slightly higher coverage than the rich.

Three countries should be highlighted in table 1. The Central African Republic (CAR) has the largest urban-rural coverage gap, of 32.8% points (p<0.001). The smallest urban-rural difference in absolute inequality was observed in Panama (−77.1 ppts; p<0.001), where urban inequality was small and rural inequality was very large. In contrast, the widest gap was observed in Chad (64.2 ppts; p<0.001), where urban inequality was marked and rural inequality was small. On average, women in the poorest urban quintile showed coverage levels that were 10.7% points (from −10.6 in Malawi to 34.0 in CAR) higher than rural women in the same quintile.

Only four countries had lower SBA coverage in urban than in rural areas (Jordan, Kosovo, Malawi, Thailand). The maximum difference was 1.3% point in Malawi, and none of these differences were statistically significant (p>0.05). On the other hand, nine countries had rural coverage at least 10% points lower than urban coverage (online supplementary table 1).

In only six countries the urban coverage in the poorest quintile was smaller than the rural coverage; and only in Malawi, the maximum difference reached 10.6% points and this difference was statistically significant (p<0.001). The difference between urban and rural SII was smaller than 10% points in 18 countries, all with p>0.05.

In most countries, rural areas were more inequitable than urban areas. Only four countries had a pro-urban difference greater than 10% points in the SII (South-Sudan, Sierra-Leone, Chad and CAR), and almost all had p value below 0.05, except for Sierra Leone (p=0.11). In contrast, 15 countries had SII in rural areas more than 10% points higher than in urban areas (all statistically significant).

Eight countries had CIX in rural areas more than 10% points higher than in urban areas (all with p<0.001), and only in Chad, CIX in urban area was more than 10% points (CIX: 14.1, p<0.001).

**Figure 1:**
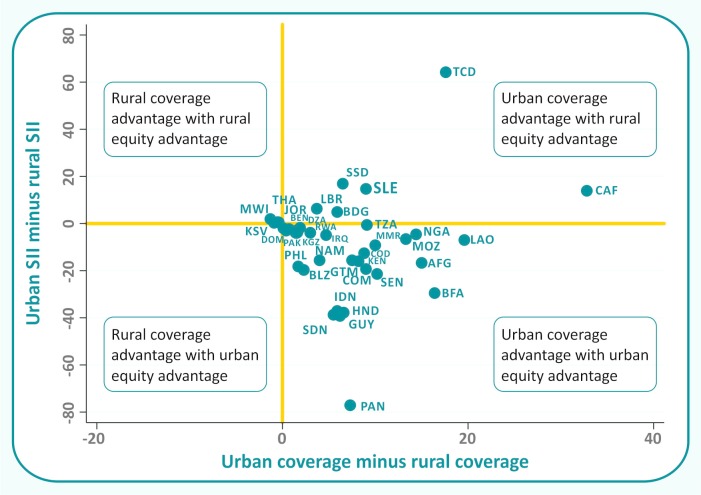
Distribution of the 37 countries according to the urban-rural differences in coverage and in absolute inequality. SII, slope index of inequality.

Figure 1 shows a plot of the urban-rural difference in the SII against the urban-rural gap in coverage, with each country represented by one dot (country codes are listed in web online supplementary appendix 1). The vertical and horizontal lines separate four quadrants. Positive values indicate that coverage or absolute inequality was higher among urban women, while negative values indicate that coverage or inequality was higher among rural women. In theory, the graph would allow the identification of four groups of countries, but if one ignores small, non-significant differences, the countries are lumped into two quadrants. The lower right quadrant, where urban areas have higher coverage and less inequality than rural areas, includes most countries. The top right quadrant, where urban areas have higher coverage and greater inequality, includes a small number of countries.

10.1136/bmjgh-2018-000898.supp2 Supplementary data

**Figure 2:**
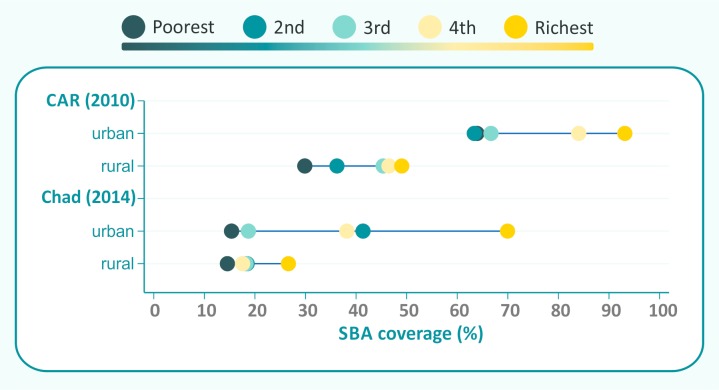
Countries with urban coverage advantage and rural inequality advantage. SBA, skilled birth attendance.

For further analyses of countries in these two quadrants, we focused on the nine countries where the difference between urban and rural coverage was greater than 10% points. Two of these countries are in the upper right quadrant in figure 1, Chad and the Central African Republic. The equiplots in figure 2 show that urban areas in both countries present top-inequality patterns, that is, the richest quintiles are well ahead of the rest of the population. Both countries also show more inequality in urban than in rural areas. The Central African Republic represents an extreme example in which all urban quintiles show higher coverage than even the wealthiest rural quintile; the difference in coverage between poorest urban and rural women reached 34% points. The two countries show very low coverage (mean of 30.3%) in rural areas as a whole.

**Figure 3:**
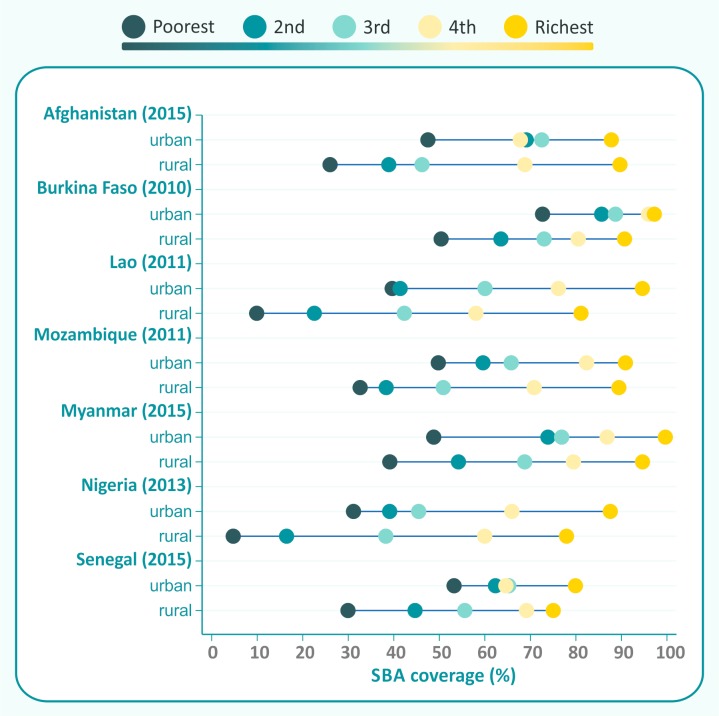
Countries with urban coverage advantage, but with similar or urban inequality advantage. SBA, skilled birth attendance.

Figure 3 shows the remaining seven countries with an urban coverage advantage of 10% points or greater; all of these are in the lower right quadrant in figure 1. In general, inequalities are wider in rural than in urban areas, but in four countries (Lao, Mozambique, Myanmar and Nigeria), the differences between the urban and rural slope indices were less than 10% points. In these countries, the average coverage among rural women in the poorest quintile is only 27.5 %, compared with 49.0% among urban women in the same quintile.

Countries where the difference between urban and rural coverage is below 10 percent points are shown in online supplementary figures 1-3.

10.1136/bmjgh-2018-000898.supp4 Supplementary data

Table 2 shows the mean values of coverage and inequality indicators according to the seven world regions. CEE & CIS includes only two countries: Kosovo and Kyrgyzstan. In these countries, both women in urban and rural areas have reached universal coverage with even the poorest rural women showing an average coverage of 97.2%, and inequalities are virtually non-existent. In the other extreme, South Asia presented the lowest average coverage in both urban and rural areas as well as in the poorest urban and rural quintiles. This region also presented the greatest socioeconomic inequalities. Last, the largest difference in absolute inequality between urban and rural areas was observed in Latin American and the Caribbean (−31.5% points), where a bottom inequality pattern is evident with the rural poorest lagging well behind all other groups, except for the Dominican Republic. A detailed presentation of results by region is available in online supplementary figure 4–10.

**Table 2:** Mean values of coverage (%) and inequality indicators for the seven world regions

Region	Urban coverage	Rural coverage	Urban CIX	Rural CIX	Urban SII	Rural SII	Coverage in urban poorest	Coverage in rural poorest
CEE & CIS	99.1	98.8	0.3	0.4	1.1	2.75	97.6	97.2
East Asia & Pacific	81.9	74.5	6.6	17.4	35.0	48.4	61.9	50.3
Eastern & Southern Africa	76.0	69.6	7.3	12.8	27.0	32.6	61.0	53.6
Latin American & Caribbean	94.4	89.3	2.0	9.6	11.0	42.5	87.4	71.2
Middle East & North Africa	93.0	90.3	1.5	4.8	8.6	19.8	83.0	80.3
South Asia	53.0	51.2	12.6	21.5	47.0	51.7	33.3	26.0
West & Central Africa	70.0	57.4	8.1	13.2	36.0	32.3	54.6	39.8

CIX, concentration index; SII, slope index of inequality.

**Figure 4:**
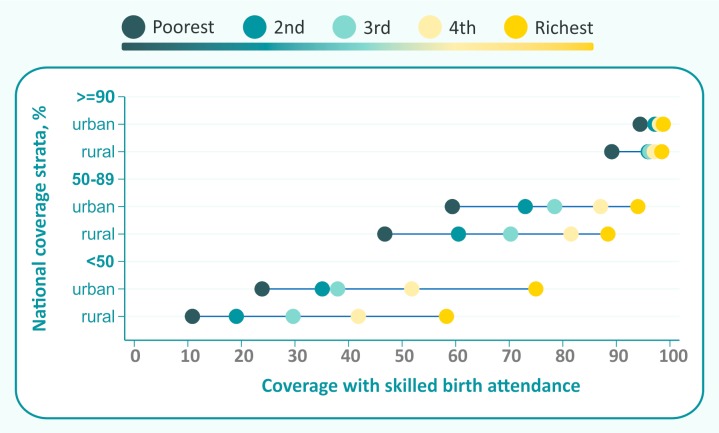
Average coverage by wealth quintile and place of residence in countries with different levels of national SBA coverage. SBA, skilled birth attendance.

**Figure 5:**
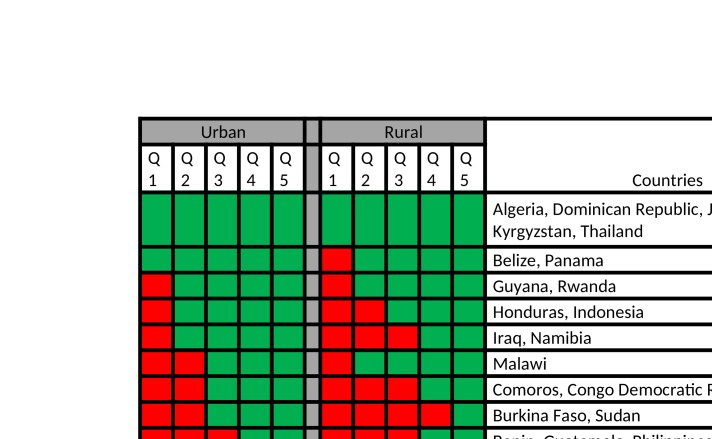
Countries with SBA coverage lower than 90% (red cells) according to wealth quintiles index in urban and rural areas. SBA, skilled birth attendance.

Figure 4 shows average coverage levels for three country groups, classified according to national coverage strata. In countries with national coverage above 90%, only the poorest rural women lag behind, showing a bottom inequality pattern. In countries with national coverage between 50% and 90%, average coverage by quintile is spread evenly, with urban quintiles showing higher coverage than the corresponding rural quintiles. Coverage gaps are wide, with greater than 90% average coverage in the richest urban quintiles, compared with less than 50% in the poorest rural quintiles. In the third group—countries with less than 50% national coverage—top inequality patterns predominate in both urban and rural areas, with coverage in the richest quintiles being much higher than for the rest of the population. Again, the gaps are wide, from an average value of 75% in the richest urban quintiles and only 10% in the poorest rural quintiles.

Figure 5 summarises our findings, by classifying countries according to which subgroups have coverage below 90%, which constitutes one of the targets of SDG 2030. Six countries (Algeria, Dominican Republic, Jordan, Kosovo, Kyrgyzstan, Thailand) have reached 90% coverage in all quintiles in both urban and rural areas. At the other extreme, none of the quintiles from Bangladesh, Pakistan, Sierra Leone, South Sudan, Chad, Nigeria, Senegal have reached 90% universal coverage.

## Discussion

This multicountry analysis of population-based surveys was carried out to assess socioeconomic inequalities in delivery care for urban and rural women separately, within countries as well as across countries and regions of the world. In particular, we were interested in studying whether the poorest urban women in different countries have an advantage over the poorest rural women in terms of access to SBA.

Our findings show that in 33 out of the 37 countries with data, rural coverage was lower than urban coverage. Only four countries had higher urban coverage, but none of these differences were statistically significant. A second important finding was that poorest rural women were almost always worse off compared with the poorest urban women. In only six countries, the poorest urban women had lower coverage than the poorest rural women, but none of these differences were significant. Besides, in most countries studied, socioeconomic inequalities in rural areas were larger than in urban areas. Our analyses (figure 1) show that most countries presented a double urban advantage, that is higher coverage and narrower inequalities in urban than in rural areas. This figure is based on the WHO recommendation for using four-quadrant plots for reporting on inequality according to national coverage.^17^

A second approach to classifying countries was based on seven regions of the world. Three regions (CEE & CIS, Latin American & the Caribbean, Middle East & North Africa) had high coverage in groups. In the Middle East & North Africa, more efforts are necessary for both urban and poor rural women in Sudan to reach universal coverage and reduce inequalities (Figures R1 and R5). In Latin American and the Caribbean (Figure R4), greater efforts are especially needed to target the poorest rural women that were lagging behind. South Asia (Figure R6) was the region with the lowest average in coverage and greatest inequalities compared with the other regions, and policies to increase coverage should be addressed to the entire population, except perhaps for the richest women in urban and rural areas in a couple of countries. In the other three regions (Figures R2, R3 and R7), no clear regional patterns were observed, policy actions should be implemented by taking into account the coverage level of each country individually.

When analyses were stratified according to national coverage (figure 4), the group with coverage equal or greater than 90%, presented similar pattern to that observed in Latin America and the Caribbean, showing the need to target interventions at the rural poor. In contrast, when coverage level was lower than 50%, almost all subgroups had low coverage, and great effort should be deployed to promote rapid uptake by the whole population by identifying and addressing barriers to utilisation. Our results are consistent with a recent set of analyses of institutional deliveries coverage in 286 national surveys from 89 LMICs, which was restricted to national coverage by quintile, rather than the intersectionality between wealth and residence. The study evidenced that at low national coverage, top inequality is observed with coverage in the wealthiest quintile taking off rapidly. In contrast, when national coverage is high, bottom inequality became evident with the poorest quintile lagging behind.^8^

Many studies in the literature address coverage with delivery care interventions according to wealth or to place of residence.^9 18–20^ However, few studies have focused their attention on coverage according to wealth separately in urban and rural dwellers. In most countries, rural women are concentrated in the poorest wealth quintiles so that wealth and residence interact. Furthermore, many of the earlier analyses relied on asset indices that were biased to urban dwellers. It was only in 2008 that Rutstein showed the advantage of collecting information in household assets that are specific to urban and rural dwellers and developed an approach to merge information from both areas into a single asset index.^16^ This approach was used in the present analyses.

Some earlier studies compared coverage between urban poor and rural poor families.^4 7 21^ In 2010, Matthews *et al* published an analysis of 30 countries, arguing that ‘the urban advantage’ is, for some, non-existent and that ‘the urban poor did not necessarily have better access to services than the rural poor, despite their proximity to services’.^4^ Our more recent analyses, based on a larger number of surveys, identified only one country where SBA coverage was significantly higher among the rural poor than among the urban poor (Malawi) and another five countries where urban coverage among the poor was higher, but not significantly so. Matthews *et al* suggested a typology for urban inequality: massive exclusion (when most of the population does not have access to a service) and urban marginalisation (when only the poorest are excluded). In our analyses, the only country with evidence of ‘massive exclusion’ was South Sudan, where coverage among the urban rich was below 50%; all other countries had coverage of 70% or higher among this group. In contrast, many countries showed evidence of marginalisation, or bottom inequality, both for urban and rural areas.

Another approach to a typology was proposed by Channon *et al*; examining facility births in 33 LMICs with three or more surveys over time, they proposed a common pathway to describe progress to universal health coverage, with the urban rich being the first to obtain universal coverage followed by the rural rich, the urban poor and finally the rural poor, the last to be reached.^7^ Our cross-sectional analyses are for the most part consistent with such a pattern. We only found one country where the urban poor had higher coverage than the rural rich—the Central African Republic.

In our analyses, we focused on the urban poor, rather than slum dwellers, as the latter information is not readily available from surveys. Fink *et al*, in recent analysis of 191 DHS from 73 LMICs, were able to identify slum areas, defined on the basis of the characteristics of the urban clusters where they lived.^22^ Compared with children living in non-slum urban or rural areas, children from slums had significantly better health indicators than those living in rural areas, but far worse than children in better-off neighbourhoods in the same urban settlements.^22^

Our analyses were focused on coverage. Higher coverage among urban poor, however, does not necessarily imply better health status, as there may be other disadvantages of living in urban slums—related to crowding and poor sanitation, for example—that may outweigh the benefit of greater access to services. For example, Madise *et al* found increased infant mortality among the urban poor in Zambia, compared with rural poor.^5^

Our study had some limitations. The first is related to sample sizes. Of the 88 surveys analysed, 9 were excluded as there were no women in at least 1 of the 10 cells of the combination of wealth quintiles and urban/rural areas. Another 42 surveys were partially analysed because at least one cell included fewer than 25 women, resulting in lack of precision of the estimates. Only 37 surveys (42%) had 25 or more observations in all ten cells. This proportion was higher (15/26 or 57.7%) in countries with more than 70% rural population, intermediate (17/39 or 43.6%) in those with 40%–70% rural population and lower (5/23 or 21.7%) in countries with less than 40% rural population. This is because the absolute number of observations tends to be low in the richest quintile in rural areas, in countries where most of the population is urban.

Second, area of residence and wealth asset indices were assessed at the time of the surveys, whereas SBA coverage refers to births up to 2 years (MICS) or 3 years (DHS) before the surveys. It is possible, therefore that some women may have changed categories since the birth took place. However, we believe that few women would change status in this short period of time, and this would have very little effect on the estimates.

Third, our analyses are based on women self-report of SBA indicator, which has been criticised due to difficulties of women in discriminating accurately among the types of birth attendants.^23^ However, previous studies conducted in Kenya and Mexico have shown that this indicator can be used to generate acceptable estimate of SBA coverage at population level^14 15^ and a recent global analysis found a very high correlation between SBA coverage and the proportion of institutional deliveries.^24^

Fourth, our study was focused in only two keys dimension of inequality: wealth asset indices and place of residence. Other important aspects of inequality in coverage such as distance to health facility, religion, ethnicity, differ between countries and region of the world, but could not be assessed. Distance to health facilities will be smaller in urban than in rural areas, and referral systems are more likely to result in access to SBAs and emergency obstetric services if needed.

Fifth, some surveys included in our study are from countries with ongoing conflict or humanitarian crisis, such as Afghanistan, Iraq, Pakistan, South Sudan, Sudan, Central African Republic, and this could affect both coverage and inequalities.

Finally, it should be highlighted that SBA coverage does not necessarily ensure high-quality care, nor access to emergency obstetric care and life-saving commodities. Such aspects have not been evaluated in the survey studied.

Despite these limitations, some positive points should be highlighted. This study was based on large cross-sectional surveys that are nationally representative and comparable within and across countries. Such surveys include DHS and MICS conducted by national agencies with economic and technical assistance from internationally renowned organisations such as USAID, Unicef, UNFPA and the World Bank.

Second, our analysis was disaggregated by wealth quintile indices in urban and rural areas separately. This allows us to assess inequalities in coverage between subgroups, identify the subgroups where special attention is needed in both urban and rural residents and to assess the gap between urban and rural poorest women.

Finally, this analysis represents an important step as it can be used to guide policymakers’ decisions about which subgroups in each country may require special efforts to increase SBA coverage, an indicator that has received high visibility in the MDS and SDG eras, as an essential component of achieving progress towards maternal and newborn health goals.

## Conclusion

This analysis shows the presence and magnitude of twofold, double urban advantage: the urban advantage of higher urban coverages and the urban advantage of lower urban wealth-related inequalities. It also identifies countries where poor rural women are most disadvantaged in comparison with urban women. Such analysis represents an important step as it may guide policy decisions about which subgroups require special efforts and on whether targeting should be employed to increase SBA coverage.

10.1136/bmjgh-2018-000898.supp3 Supplementary data
