# Supplementary material for: Socioeconomic inequalities in access to skilled birth attendance among urban and rural women in low-income and middle-income countries
Source: BMJ Glob Health. 2018 Dec 1;3(6):e000898. doi: 10.1136/bmjgh-2018-000898 (PMC6278921; doi:10.1136/bmjgh-2018-000898)
Supplement: Supplementary data [file bmjgh-2018-000898supp001.pdf]

Supplementary Table S1: Indicators of coverage and inequality in the 79 countries.

Country-Year	Place of residence	SBA (%)	Coverage (%) and number of deliveries by wealth quintile					SII	CIX	P-value for urban minus rural women	P-value for urban minus rural poorest	P-value for SII urban minus SII rural	P-value for CIX urban minus CIX rural	Interaction p-value
			Poorest	Second	Third	Fourth	Richest							
Afghanistan 2015	urban	68.9	47.5 (291)	69.1 (238)	72.5(344)	67.8 (1315)	87.8 (2540)	36.6	7.3	p=0.007	p<0.001	p=0.016	p<0.001	p<0.001
	rural	53.9	26.0 (3156)	38.9 (4237)	46.2 (4025)	68.8 (2965)	89.7 (280)	53.3	21.0					
Algeria 2012	urban	97.7	97.5 (339)	96.9 (673)	97.7 (938)	97.8 (1011)	98.6 (859)	1.7	0.3	p=0.20	p=0.003	p=0.42	p=0.13	p<0.001
	rural	95.8	93.5 (1073)	96.3 (579)	94.0 (300)	95.4 (150)	100.0 (56)	3.5	0.8					
Armenia 2010	urban	99.8	100.0 (16)	99.7 (91)	100.0 (158)	99.1 (202)	100.0 (137)	0.1	0.1	p=0.65	p=1.00	p=0.76	p=0.08	p=1.00
	rural	100.0	100.0 (149)	100.0 (115)	100.0 (45)	100.0 (21)	100.0 (4)	0.0	0.0					
Bangladesh 2014	urban	47.1	18.4 (130)	41.6 (118)	47.1 (192)	51.8 (424)	76.8 (644)	57.0	18.6	p=0.18	p=0.86	p=0.37	p=0.003	p=0.21
	rural	41.2	17.9 (883)	28.9 (785)	37.3 (711)	52.0 (558)	70.0 (289)	52.1	25.8					
Barbados 2012	urban	98.5	100.0 (22)	92.3 (18)	100.0 (16)	100.0 (15)	100.0 (14)	3.3	0.4	p=0.73	p=1.00	p=0.09	p=0.17	p=0.999
	rural	100.0	100.0 (11)	100.0 (11)	100.0 (13)	100.0 (12)	100.0 (14)	0.0	0.0					

Belarus 2012	urban	99.9	100.0 (44)	100.0 (120)	99.7 (190)	100.0 (264)	100.0 (371)	0.2	0.0	p=0.84	p=1.00	p=0.27	p=0.26	p=1.00
	rural	100.0	100.0 (133)	100.0 (125)	100.0 (46)	100.0 (23)	100.0 (8)	0.0	0.0					
Belize 2011	urban	98.6	100.0 (25)	98.2 (52)	97.3 (61)	99.0 (60)	98.5 (50)	-0.1	-0.1	p=0.42	p=0.001	p=0.012	p=0.004	p=0.03
	rural	96.3	87.5 (188)	98.6 (99)	97.9 (72)	100 (66)	97.3 (30)	19.7	3.2					
Benin 2011	urban	85.7	64.5 (389)	80.6 (286)	87.5 (380)	97.3 (814)	98.8 (1107)	34.3	5.3	p=0.78	p=0.70	p=0.70	p=0.038	p=0.78
	rural	85.0	66 (1396)	79.3 (1400)	85.1 (1268)	95.8 (704)	99 (128)	36.6	7.6					
Bhutan 2010	urban	79.2	100.0 (1)	31.1 (4)	85.8 (70)	84.3 (218)	94.9 (264)	18.9	3.8	p=0.007	p<0.001	p<0.001	p<0.001	p<0.001
	rural	62.6	34.1 (510)	43.2 (529)	63.1 (457)	76.8 (277)	95.7 (265)	61.2	20.5					
Bosnia & Herz. 2011	urban	100.0	100.0 (12)	100.0 (22)	100.0 (38)	100.0 (560	100.0 (115)	0.0	0.0	p=0.55	p=1.00	p=0.23	p=0.23	p=1.00
	rural	99.9	100.0 (100)	99.6 (128)	100.0 (114)	100.0 (83)	100.0 (50)	0.2	0.0					
Burkina Faso 2010	urban	88.1	72.7 (71)	85.7 (99)	88.7 (180)	96 (474)	97.2 (1163)	10.7	1.8	p=0.001	p<0.001	p<0.001	p<0.001	p=0.0002
	rural	71.6	50.4 (1626)	63.5 (1779)	73 (1825)	80.5 (1491)	90.7 (294)	40.3	10.1					
Burundi 2010	urban	82.6	76.4 (18)	84.8 (13)	79.6 (14)	78.6 (25)	93.6 (329)	21.9	4.2	p<0.001	p<0.001	p=1.00	p=0.15	p=0.46
	rural	64.0	54.9 (988)	58.5 (1028)	64.1 (982)	66 (957)	76.7 (545)	21.8	5.9					
CAR 2010	urban	74.2	63.9 (135)	63.4 (164)	66.7 (301)	84 (461)	93.1 (385)	37.5	7.5	p<0.001	p<0.001	p=0.027	p=0.15	p=0.096
	rural	41.4	29.9 (900)	36.2 (964)	45.4 (784)	46.5 (357)	49 (94)	23.5	10.7					

Cambodia 2014	urban	94.5	82.2 (11)	95.1 (15)	98.4 (29)	97.5 (88)	99.1 (511)	5.2	0.7	p=0.34	p=0.34	p<0.001	p<0.001	p=0.67
	rural	92.2	79 (1037)	90.8 (831)	95.2 (841)	98 (707)	98.1 (369)	27.4	4.7					
Chad 2014	urban	36.7	15.4 (252)	41.4 (58)	18.7 (76)	38.2 (214)	69.9 (1677)	68.8	18.0	p<0.001	p=0.76	p<0.001	p<0.001	p=0.06
	rural	19.1	14.6 (1790)	18.5 (2096)	18.2 (2126)	17.6 (2040)	26.6 (221)	4.6	3.9					
Colombia 2015	urban	98.9	97.8 (516)	99.3 (1867)	99.2 (1268)	99.2 (698)	99.0 (382)	0.1	0.0	p=0.07	p<0.001	p<0.001	p<0.001	p<0.001
	rural	97.2	86.5 (1846)	99.5 (305)	100.0 (11)	100.0 (6)	100.0 (4)	51.5	7.6					
Comoros 2012	urban	92.1	82.2 (93)	86.4 (113)	94.4 (125)	99.1 (149)	98.4 (192)	20.2	3.3	p=0.020	p<0.001	p=0.009	p<0.001	p=0.13
	rural	83.1	63.9 (427)	78.6 (298)	89.6 (257)	91.7 (175)	91.8 (121)	39.5	8.4					
Congo D. R. 2013	urban	89.2	81.6 (320)	84.7 (187)	88.9 (358)	92.8 (1057)	97.8 (1479)	16.3	2.6	p=0.022	p<0.001	p=0.016	p<0.001	p=0.02
	rural	80.4	65.5 (2733)	71.9 (2333)	78 (1995)	91.6 (897)	94.8 (43)	28.9	6.4					
Costa Rica 2011	urban	99.2	99.7 (93)	99.5 (211)	99.5 (89)	97.1 (760	100 (61)	-0.6	0.0	p=0.59	p=0.048	p=0.11	p=0.05	p=0.18
	rural	97.8	95.2 (208)	98.6 (98)	100.0 (58)	100.0 (39)	95.2 (21)	7.2	1.6					
Dominican Rep. 2014	urban	98.8	98.0 (996)	99.4 (1121)	99.5 (1064)	98.3 (920)	98.9 (806)	0.2	0.1	p=0.94	p=0.90	p=0.16	p=0.27	p=0.65
	rural	98.8	97.8 (1457)	98.1 (580)	99.4 (323)	99.1 (207)	99.4 (92)	2.2	0.4					
El Salvador 2014	urban	99.0	97.6 (120)	98.6 (196)	99.6 (297)	99.5 (391)	99.5 (362)	1.8	0.3	p=0.25	p=0.013	p=0.002	p<0.001	p=0.052

	rural	97.6	92.4 (477)	97.6 (345)	98.7 (209)	99.1 (65)	100.0(19)	12.7	2.1					
Ethiopia 2011	urban	22.8	11.4 (58)	11.3 (14)	2.5 (16)	27.2 (94)	61.8 (1008)	71.4	27.0	p<0.001	p<0.001	p<0.001	p=0.64	p=0.57
	rural	7.4	2.7 (1972)	3.8 (1182)	4.1 (1079)	7.8 (986)	18.7 (292)	9.4	29.9					
Gabon 2012	urban	93.6	87.2 (648)	94.1 (683)	95.9 (428)	95.1 (363)	95.5 (257)	6.5	1.2	p=0.004	p<0.001	p<0.001	p<0.001	p<0.001
	rural	83.9	66 (1161)	78.2 (174)	77.6 (89)	97.6 (33)	100.0 (17)	33.6	9.5					
Gambia 2013	urban	73.8	72.6 (97)	74.2 (109)	66.7 (250)	73.4 (646)	82.1 (632)	17.1	3.8	p=0.001	p<0.001	p=0.002	p=0.007	p=0.77
	rural	43.7	44.9 (1161)	42.7 (1231)	38.9 (834)	33.4 (165)	58.5 (13)	-9.5	-3.8					
Ghana 2014	urban	84.2	69.4 (127)	73.1 (135)	86.2 (315)	94.5 (439)	97.6 (446)	27.0	4.6	p=0.06	p<0.001	p=0.016	p<0.001	p=0.001
	rural	75.7	48.5 (1033)	61.8 (666)	71.6 (338)	96.5 (111)	100.0 (8)	47.1	13.2					
Guatemala 2014	urban	77.8	49.2 (206)	67.4 (303)	82.0 (506)	92.9 (747)	97.6 (775)	47.4	9.0	p=0.03	p=0.008	p=0.004	p<0.001	p=0.10
	rural	70.3	38.7 (1841)	54.6 (1437)	76.9 (970)	90.9 (534)	90.5 (155)	63.0	19.5					
Guinea 2012	urban	63.9	52.2 (23)	28.2 (29)	68.7 (79)	78.6 (535)	91.6 (593)	35.3	6.5	p=0.014	p<0.001	p=0.42	p<0.001	p<0.001
	rural	48.2	19.5 (997)	28.2 (807)	38.5 (787)	54.9 (383)	100 (2)	41.8	23.0					
Guinea-Bissau 2014	urban	61.9	45.6 (35)	58.2 (76)	47.8 (180)	74.6 (292)	83.3 (236)	40.8	9.1	p=0.002	p<0.001	p=0.003	p=0.94	p=0.051
	rural	40.4	25.3 (938)	27 (683)	30.3 (569)	49.6 (167)	69.9 (20)	17.5	9.4					

Guyana 2014	urban	99.7	98.5 (41)	100.0 (53)	100.0 (52)	100.0 (56)	100.0 (56)	1.3	0.2	p=0.045	p<0.001	p<0.001	p<0.001	p<0.001
	rural	93.5	76.3 (417)	94.6 (189)	96.4 (156)	100 (116)	100.0 (122)	40.5	7.2					
Honduras 2011	urban	91.6	79.9 (115)	88.8 (274)	93.6 (580)	97.4 (719)	98.2 (607)	13.6	2.3	p=0.004	p<0.001	p<0.001	p<0.001	p<0.001
	rural	85.0	59.5 (2078)	81.7 (1293)	89.5 (638)	95.6 (297)	98.6 (123)	51.4	12.0					
Indonesia 2012	urban	90.1	75.3 (527)	90.1 (805)	92.5 (1074)	95.3 (1266)	97.3 (1265)	15.7	2.8	p=0.012	p<0.001	p<0.001	p<0.001	p<0.001
	rural	84.2	57.8 (2758)	81.1 (1420)	89.1 (904)	95.2 (580)	97.8 (313)	52.8	12.4					
Iraq 2011	urban	92.9	85.4 (1111)	91.2 (1811)	95.7 (1865)	96.2 (1623)	96 (1348)	10.1	1.7	p=0.016	p=0.009	p=0.16	p=0.009	p=0.23
	rural	88.2	80.6 (3476)	87 (1452)	87.4 (756)	90.7 (397)	95.2 (135)	15.1	3.3					
Jamaica 2011	urban	99.8	100.0 (63)	99.2 (80)	100.0 (88)	100.0 (73)	100.0 (78)	0.5	0.1	p=0.74	p=0.042	p=0.07	p=0.048	p=0.999
	rural	99.0	94.9 (93)	100.0 (60)	100.0(45)	100.0 (33)	100.0 (17)	9.7	1.2					
Jordan 2012	urban	99.5	98.4 (1119)	100.0 (994)	100.0 (904)	99.1 (765)	100.0 (393)	1.2	0.2	p=0.67	p=0.31	p=0.69	p=0.72	p=0.35
	rural	99.9	99.4 (512)	99.9 (660)	100.0 (488)	100.0 (223)	100.0 (49)	0.7	0.1					
Kazakhstan 2015	urban	99.4	100 (75)	98.5 (82)	99.6 (257)	99.2 (363)	99.7 (355)	0.4	0.0	p=0.89	p=0.14	p=0.32	p=0.33	p=0.33
	rural	99.3	99.6 (275)	99.2 (285)	99.3 (215)	98.2 (61)	100.0 (14)	-0.7	-0.2					
Kenya 2014	urban	72.0	48 (565)	66 (533)	66.1 (542)	86 (942)	93.8 (1491)	44.0	8.3	p=0.005	p<0.001	p<0.001	p<0.001	p<0.001

	rural	63.8	32.6 (3713)	52.2 (2028)	66.7 (1527)	82.2 (884)	85.2 (218)	60.1	20.9					
Kosovo 2013	urban	98.6	95.2 (29)	100.0(24)	100.0 (42)	97.6 (56)	100.0 (86)	3.0	0.6	p=0.74	p=0.27	p=0.92	p=0.36	p=0.46
	rural	99.4	98.2 (113)	99.0 (107)	100.0 (85)	100.0 (67)	100.0 (28)	2.8	0.3					
Kyrgyzstan 2014	urban	99.6	100.0 (46)	100.0 (39)	100.0 (106)	99.0 (193)	99.2 (237)	-0.9	-0.1	p=0.25	p=0.040	p=0.23	p=0.22	p=0.999
	rural	98.1	96.2 (354)	98.8 (344)	98.6 (273)	98 (145)	98.9 (29)	2.7	0.5					
Lao 2011	urban	62.4	39.6 (35)	41.4 (87)	60.0 (137)	76.2 (240)	94.6 (391)	59.2	12.8	p<0.001	p<0.001	p=0.13	p<0.001	p<0.001
	rural	42.8	9.9 (1332)	22.5 (923)	42.3 (711)	58 (428)	81.1 (160)	66.1	38.8					
Lesotho 2014	urban	82.3	100.0 (1)	55.6 (26)	66.5 (81)	95.0 (150)	94.6 (221)	30.9	5.2	p=0.79	p<0.001	p=0.69	p=0.10	p<0.001
	rural	80.8	65.1 (519)	73.1 (397)	86.1 (352)	86 (207)	93.8 (78)	33.7	7.6					
Liberia 2013	urban	69.2	60.6 (155)	54.5 (225)	63.6 (443)	78.6 (394)	88.8 (242)	40.2	9.4	p=0.56	p=0.001	p=0.48	p=0.41	p=0.23
	rural	65.5	44.1 (1484)	56.9 (1017)	64.1 (494)	75.6 (109)	86.9 (32)	33.9	11.3					
Macedonia 2011	urban	98.2	100.0 (15)	93.2 (33)	100.0 (56)	100.0 (73)	97.7 (87)	1.3	0.3	p=0.85	p=0.16	p=0.62	p=0.41	p=0.245
	rural	99.0	97.2 (78)	97.7 (66)	100.0 (51)	100.0 (31)	100.0 (13)	4.3	0.5					
Malawi 2015	urban	89.5	77.2 (68)	86.8 (87)	92.7 (117)	94.7 (304)	96.2 (1055)	8.3	1.6	p=0.64	p<0.001	p=0.55	p=0.43	p=0.61
	rural	90.8	87.8 (2275)	90.0 (2158)	89.7 (1836)	92.5 (1541)	94.1 (741)	6.3	1.2					

Mali 2012	urban	61.5	0.0 (2)	61.4 (9)	59.2 (26)	90.7 (397)	96.3 (1092)	17.4	2.4	p=0.85	p<0.001	p<0.001	p<0.001	p=0.632
	rural	60.1	37.9 (1148)	47.2 (1190)	52.5 (1168)	73.9 (779)	88.8 (205)	46.0	15.1					
Mauritania 2011	urban	76.4	56.7 (12)	62.7 (72)	76.2 (221)	90.6 (451)	95.9 (606)	31.8	5.6	p=0.022	p<0.001	p<0.001	p<0.001	p=0.003
	rural	64.4	26.2 (771)	43.8 (708)	69 (482)	86.2 (256)	96.9 (50)	69.2	25.7					
Mexico 2015	urban	98.9	96.5 (227)	99.5 (473)	99.7 (416)	99.2 (352)	99.8 (214)	2.4	0.4	p=0.37	p=0.023	p<0.001	p<0.001	p=0.12
	rural	97.7	89.3 (580)	100 (215)	99.2 (103)	100.0 (49)	100.0 (13)	34.0	4.8					
Moldova 2012	urban	99.7	100.0 (13)	100.0 (22)	100.0 (34)	99 (85)	99.6 (251)	-0.1	0.0	p=0.68	p=0.15	p=1.00	p=0.85	p=0.999
	rural	98.2	97.4 (71)	100.0 (95)	100.0 (810	100.0 (48)	93.6 (23)	0.0	0.1					
Mongolia 2013	urban	99.0	100.0 (44)	99.5 (274)	98.7 (315)	98.3 (361)	98.6 (427)	-1.0	-0.1	p=0.78	p<0.001	p=0.036	p=0.045	p<0.001
	rural	98.7	97 (527)	99.3 (185)	97.4 (152)	100.0 (65)	100.0 (22)	3.3	0.5					
Montenegro 2013	urban	99.2	100.0 (24)	100.0 (54)	98.0 (81)	97.9 (93)	100.0 (65)	-0.4	0.0	p=0.85	p=0.24	p=0.32	p=0.13	p=1.00
	rural	99.7	98.4 (56)	100.0 (49)	100.0 (20)	100.0 (12)	100.0 (40)	2.3	0.5					
Mozambique 2011	urban	69.7	49.8 (126)	59.6 (1000	65.8 (208)	82.3 (527)	90.9 (1293)	42.4	8.7	p=0.003	p<0.001	p=0.27	p<0.001	p=0.001
	rural	56.4	32.6 (1090)	38.3 (1230)	50.9 (1173)	70.8 (984)	89.5 (206)	48.9	18.1					
Myanmar 2015	urban	77.2	48.8 (36)	73.9 (46)	76.9 (83)	86.9 (197)	99.7 (258)	47.1	8.0	p=0.09	p=0.07	p=0.004	p<0.001	p=0.40

	rural	67.2	39.1 (794)	54.2 (580)	68.8 (467)	79.5 (311)	94.6 (95)	56.3	17.9					
Namibia 2013	urban	92.0	77.3 (39)	96.9 (216)	91.1 (320)	96.4 (459)	98.4 (374)	7.8	1.5	p=0.22	p=0.68	p=0.001	p<0.001	p<0.001
	rural	88.0	75.7 (648)	85.1 (486)	87.7 (387)	92.8 (199)	98.7 (33)	23.3	5.1					
Nepal 2014	urban	78.6	58.5 (16)	80.0 (18)	69.9 (17)	84.7 (62)	99.8 (149)	44.7	6.8	p<0.001	p<0.001	p=0.002	p<0.001	p<0.001
	rural	55.8	24.3 (438)	43.5 (418)	55.1 (424)	68.6 (338)	87.6 (167)	62.6	22.4					
Niger 2012	urban	68.9	17.8 (5)	100 (2)	69.2 (16)	69.1 (204)	88.3 (1483)	31.8	6.1	p<0.001	p=0.07	p=0.32	p<0.001	p<0.001
	rural	27.1	14 (1321)	22.1 (1321)	23.9 (1387)	28.5 (1304)	47.2 (473)	25.7	18.5					
Nigeria 2013	urban	53.9	31.2 (223)	39.1 (444)	45.5 (1073)	65.9 (1966)	87.6 (2562)	61.1	16.0	p<0.001	p<0.001	p=0.32	p<0.001	p<0.001
	rural	39.4	4.7 (3878)	16.4 (3979)	38.2 (2700)	60.0 (1537)	77.9 (507)	65.7	47.9					
Pakistan 2012	urban	58.9	34.1 (153)	50.4 (307)	52.7 (546)	71.4 (766)	85.9 (1099)	46.7	11.8	p=0.92	p=0.98	p=0.69	p=0.018	p=0.32
	rural	58.5	34 (1396)	42.9 (1073)	56.2 (787)	74.3 (522)	85.2 (200)	49.8	17.7					
Panama 2013	urban	99.6	98.8 (86)	99.4 (148)	99.8 (221)	100 (214)	100.0 (96)	1.1	0.2	p<0.001	p<0.001	p<0.001	p<0.001	p<0.001
	rural	92.3	67.3 (1093)	98.4 (270)	100.0 (79)	95.7 (39)	100.0 (32)	78.3	15.1					
Peru 2012	urban	92.2	73.5 (156)	90.9 (844)	97.5 (1084)	99.5 (767)	99.5 (508)	16.4	2.5	p=0.16	p=0.006	p<0.001	p<0.001	p=0.05
	rural	87.6	63.4 (1448)	85.9 (733)	97.4 (107)	91.2 (20)	100.0 (1)	51.0	13.3					

Philippines 2013	urban	80.4	49.4 (218)	73.9 (329)	86.2 (421)	95.4 (448)	97.3 (325)	47.7	9.1	p=0.57	p=0.36	p<0.001	p<0.001	p=0.88
	rural	78.7	45.7 (1123)	74.4 (606)	85 (414)	92.3 (223)	96.3 (150)	65.9	17.9					
Rwanda 2014	urban	94.8	88.1 (97)	94.3 (71)	98.3 (63)	95.2 (125)	97.9 (739)	7.1	1.2	p=0.15	p=0.33	p=0.13	p=0.029	p=0.31
	rural	91.8	86 (1075)	90.9 (933)	91.7 (818)	93.6 (712)	96.8 (227)	11.0	2.2					
Sao Tome & P. 2014	urban	94.5	89.3 (108)	96.9 (79)	92.4 (93)	95.7 (86)	98.1 (86)	8.6	1.3	p=0.29	p=0.07	p=0.09	p=0.037	p=0.31
	rural	89.5	79.5 (94)	87.6 (86)	92 (59)	97.1 (55)	91.3 (12)	21.8	4.1					
Senegal 2015	urban	65.1	53.2 (80)	62.3 (136)	65.3 (229)	64.6 (375)	79.9 (241)	24.6	6.6	p=0.11	p<0.001	p=0.009	p<0.001	p=0.03
	rural	54.9	29.9 (1294)	44.7 (976)	55.6 (571)	69.2 (196)	75 (66)	45.9	17.6					
Serbia 2014	urban	98.9	100.0 (16)	100.0 (20)	100.0 (43)	99.8 (64)	94.5 (84)	-9.9	-2.0	p=0.89	p=0.023	p=0.13	p=0.09	p=0.999
	rural	99.3	96.4 (35)	100.0 (42)	100.0 (39)	100.0 (20)	100.0 (17)	4.3	0.7					
Sierra Leone 2013	urban	68.3	48.3 (84)	56.5 (62)	71.9 (201)	78.6 (817)	86.1 (972)	26.2	5.8	p=0.23	p=0.27	p=0.11	p=0.37	p=0.22
	rural	59.3	53.5 (1557)	55 (1354)	55.8 (1255)	65.2 (728)	66.9 (65)	11.5	3.7					
South Sudan 2010	urban	23.6	14.7 (98)	12.0 (89)	18.5 (173)	26.9 (238)	46.0 (398)	46.8	26.6	p=0.06	p=0.33	p=0.009	p=0.15	p=0.65
	rural	17.2	7.3 (529)	9.0 (586)	12.2 (532)	21.6 (521)	35.7 (352)	29.9	33.5					
Saint Lucia 2012	urban	100.0	100.0 (9)	100.0 (11)	100.0 (7)	100.0 (2)	100.0 (4)	0.0	2.9	p=0.83	p=0.27	p=0.37	p=0.06	p=0.999
	rural	98.4	91.8 (12)	100.0 (15)	100.0 (15)	100.0 (15)	100.0 (8)	9.5	1.0					

State of Palestine 2014	urban	99.6	99.7 (656)	99.2 (477)	99.6 (453)	100 (468)	99.3 (381)	-0.1	0.0	p=0.73	p=0.16	p=0.92	p=0.43	p=0.32
	rural	99.8	100.0 (15)	100.0 (50)	99.4 (160)	99.4 (151)	100.0 (80)	0.0	-0.1					
Sudan 2014	urban	82.9	50.6 (47)	81.9 (170)	91 (414)	94 (434)	96.9 (438)	21.3	3.6	p=0.13	p=0.52	p<0.001	p<0.001	p=0.03
	rural	77.4	47.6 (1197)	68.6 (1364)	80.7 (878)	95 (478)	95.2 (265)	60.0	14.9					
Suriname 2010	urban	96.5	96.2 (86)	93.3 (149)	98 (164)	98.8 (143)	96 (126)	3.2	0.4	p=0.43	p<0.001	p<0.001	p<0.001	p<0.001
	rural	94.3	80.2 (254)	94.9 (62)	98.4 (36)	97.8 (24)	100.0 (15)	44.3	7.1					
Swaziland 2014	urban	89.9	76.1 (6)	96.2 (20)	86 (31)	97.4 (48)	93.6 (86)	5.1	1.1	p=0.88	p=0.96	p<0.001	p=0.002	p=0.031
	rural	89.1	76.4 (244)	85.9 (219)	91.3 (189)	93.2 (97)	98.8 (47)	25.9	5.2					
Tajikistan 2012	urban	93.2	90.9 (21)	96.4 (50)	91.1 (90)	91.5 (184)	96.3 (621)	6.4	1.1	p=0.19	p<0.001	p<0.001	p<0.001	p<0.001
	rural	89.0	73.4 (531)	86.3 (563)	91.7 (549)	95.5 (445)	98.3 (141)	30.0	5.6					
Tanzania 2015	urban	74.4	50.7 (79)	68.0 (43)	72.3 (96)	84.5 (487)	91.3 (548)	40.9	7.3	p=0.11	p=0.06	p=0.92	p=0.016	p=0.86
	rural	65.4	43.0 (1349)	52.3 (1240)	61.2 (1115)	77.2 (843)	77.2 (204)	41.5	13.0					
Thailand 2012	urban	99.1	96.2 (125)	100.0 (190)	100.0 (311)	99.6 (384)	99.9 (464)	1.6	0.3	p=0.55	p=0.06	p=0.62	p=0.65	p=0.31
	rural	99.7	99.1 (215)	99.5 (277)	100.0 (333)	99.7 (303)	100.0 (160)	0.9	0.2					
Togo 2013	urban	72.1	45.6 (7)	58.0 (10)	68.2 (62)	92.5 (519)	96.1 (631)	18.7	3.3	p=0.17	p<0.001	p<0.001	p<0.001	p=0.01

	rural	60.6	27.7 (1233)	38.7 (857)	57.7 (767)	79.1 (150)	100.0 (10)	51.4	19.9					
Tunisia 2011	urban	99.3	97.3 (49)	100 (112)	100 (139)	99.8 (203)	99.6 (143)	0.5	0.1	p=0.56	p=0.11	p<0.001	p<0.001	p=0.27
	rural	98.5	93 (253)	99.6 (138)	100 (57)	100 (31)	100 (10)	15.7	2.4					
Uganda 2011	urban	82.3	85.5 (16)	69.5 (26)	80.9 (37)	83 (86)	92.4 (530)	19.9	2.6	p<0.001	p<0.001	p=0.006	p<0.001	p<0.001
	rural	58.8	43.9 (1089)	50.9 (1013)	55.4 (949)	59 (798)	84.6 (360)	31.8	10.2					
Vietnam 2013	urban	96.6	84.1 (20)	100.0 (37)	100.0 (62)	98.9 (106)	100.0 (203)	5.2	0.7	p=0.41	p=0.08	p<0.001	p<0.001	p=0.06
	rural	93.5	72.6 (274)	96.4 (251)	99.6 (230)	99.1 (208)	100.0 (73)	42.4	6.9					
Yemen 2013	urban	67.0	57.3 (16)	65.6 (74)	60.2 (212)	68.3 (881)	83.7 (1017)	31.6	7.3	p=0.002	p<0.001	p=0.003	p<0.001	p<0.001
	rural	46.5	20.1 (2124)	31.5 (2156)	44.3 (1893)	59.6 (1043)	76.8 (378)	48.3	23.5					
Zambia 2013	urban	81.2	67 (23)	69.4 (111)	82.6 (438)	91.2 (1041)	95.9 (1084)	21.9	3.9	p<0.001	p<0.001	p=0.92	p=0.006	p=0.12
	rural	62.7	48.6 (1870)	54.7 (1740)	58.5 (1180)	74.4 (345)	77.5 (74)	22.2	7.0					

Values in red show cells with a denominator below 25 observations.
