# Supplementary material for: Socioeconomic inequalities in access to skilled birth attendance among urban and rural women in low-income and middle-income countries
Source: BMJ Glob Health. 2018 Dec 1;3(6):e000898. doi: 10.1136/bmjgh-2018-000898 (PMC6278921; doi:10.1136/bmjgh-2018-000898)
Supplement: Supplementary data [file bmjgh-2018-000898supp002.pdf]

Web Appendix A: ISO codes of the 37 countries.

ISO codes	Country	ISO codes	Country
AFG	Afghanistan	KGZ	Kyrgyzstan
DZA	Algeria	LAO	Lao
BGD	Bangladesh	LBR	Liberia
BLZ	Belize	MWI	Malawi
BEN	Benin	MOZ	Mozambique
BFA	Burkina Faso	MMR	Myanmar
CAF	CAR	NAM	Namibia
TCD	Chad	NGA	Nigeria
COM	Comoros	PAK	Pakistan
COD	Congo Democratic Republic	PAN	Panama
DOM	Dominican Republic	PHL	Philippines
GTM	Guatemala	RWA	Rwanda
GUY	Guyana	SEN	Senegal
HND	Honduras	SLE	Sierra Leone
IDN	Indonesia	SSD	South Sudan
IRQ	Iraq	SDN	Sudan
JOR	Jordan	TZA	Tanzania
KEN	Kenya	THA	Thailand
XKX	Kosovo		
