# Supplementary material for: Socioeconomic inequalities in access to skilled birth attendance among urban and rural women in low-income and middle-income countries
Source: BMJ Glob Health. 2018 Dec 1;3(6):e000898. doi: 10.1136/bmjgh-2018-000898 (PMC6278921; doi:10.1136/bmjgh-2018-000898)

Supplementary Figure 1: Countries with similar urban-rural coverage and inequality.

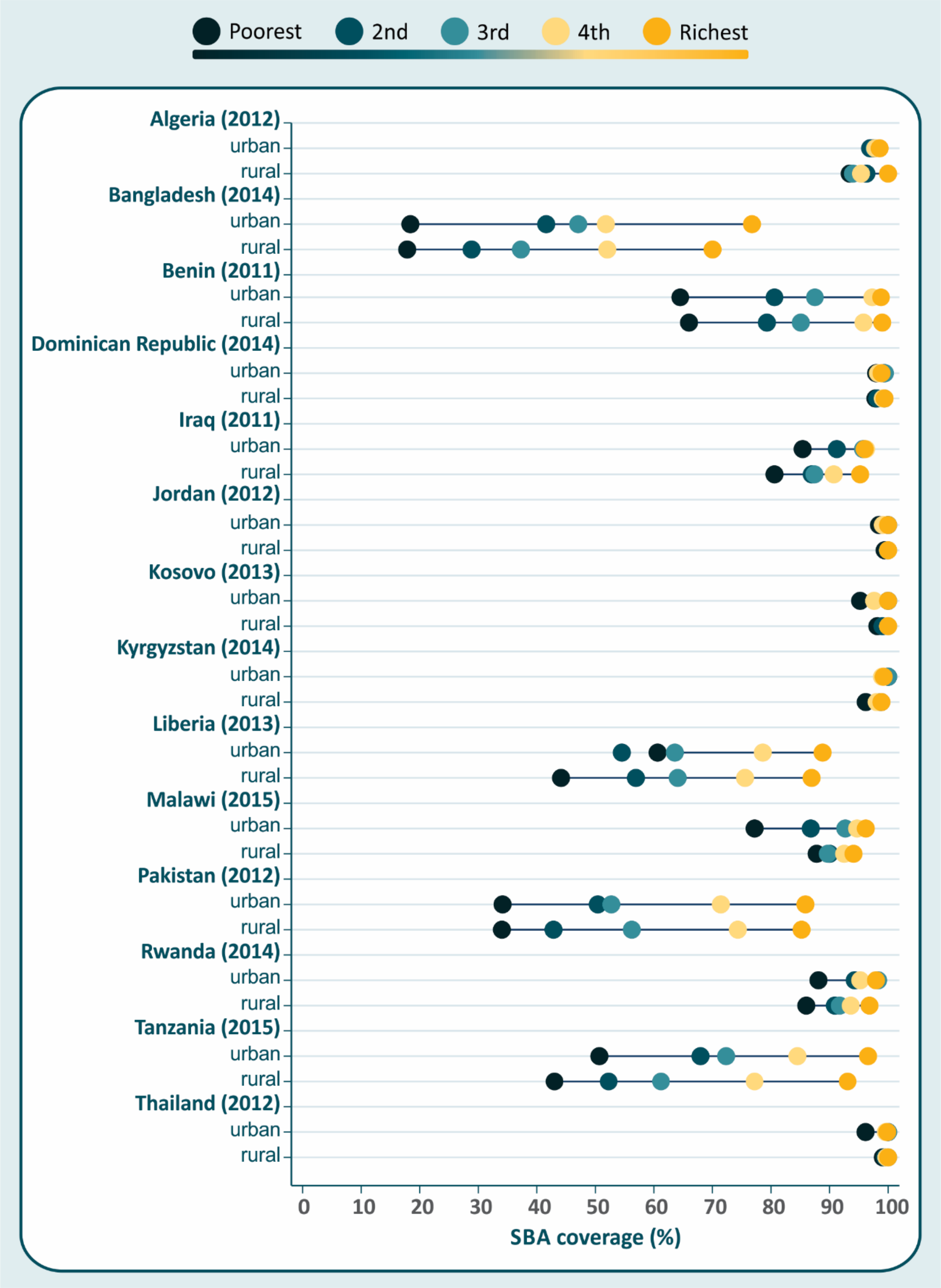

In Figure 1 countries had similar urban-rural coverage and inequality. Note that Malawi, where the difference between the urban and rural SII was only 1.9 percent point, apparently shows greater inequality in urban than rural areas. This is because the

SII takes into account the whole wealth distribution using a population-weighted regression equation, and the outlying group in Figure 1b, the poorest urban quintile, only includes 68 out of a national sample of 10,208 women. Countries in this group tended to have high national coverage, with a mean of 86.1% in urban and 84.2% in rural areas. Coverage in poorest rural women in average is 73.6, compared to 76.3 in poorest urban women.

Supplementary Figure 2: Countries with similar urban-rural coverage but with rural equity advantage.

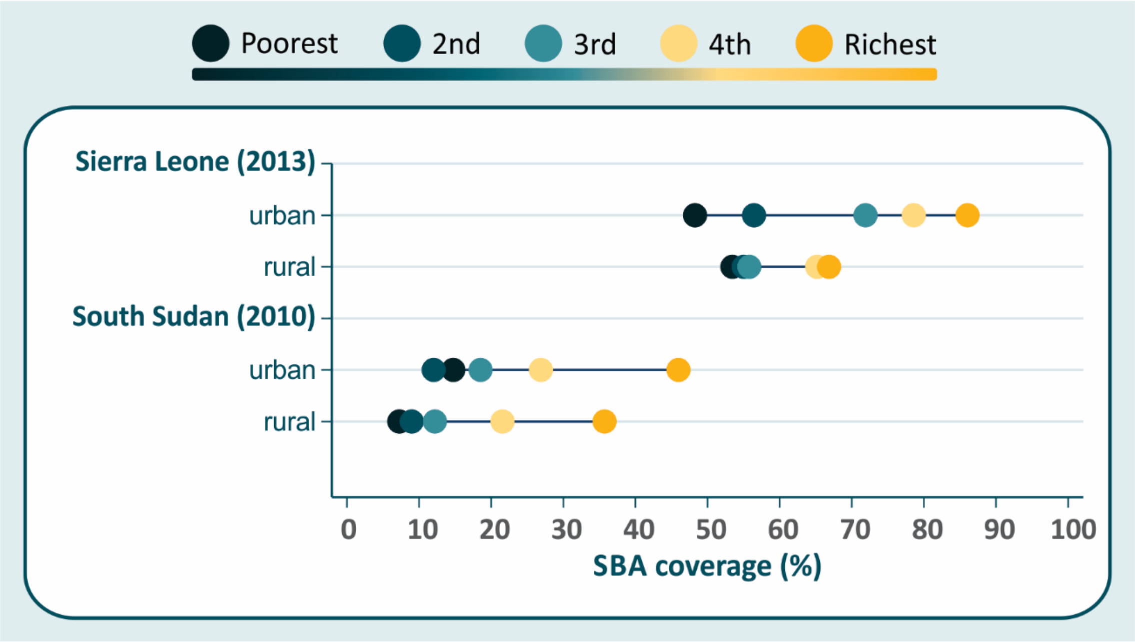

Figures 2 includes only two countries with low national coverage, where urban and rural coverage was similar but with advantage rural equity: Sierra Leone and South Sudan. In South Sudan, the Figure suggests that inequalities are similar in both areas, but as for Malawi this is due to small numbers of women in the wealthy rural and poor urban quintiles (the SII is 16.9 percent points higher in urban than in rural areas). Coverage in rural and urban poorest women in South Sudan is 7.3% and 14.7% respectively; while in Sierra Leone, it is 53.5 and 48.3% respectively.

Supplementary Figure 3: Countries with similar urban-rural coverage but with urban equity advantage.

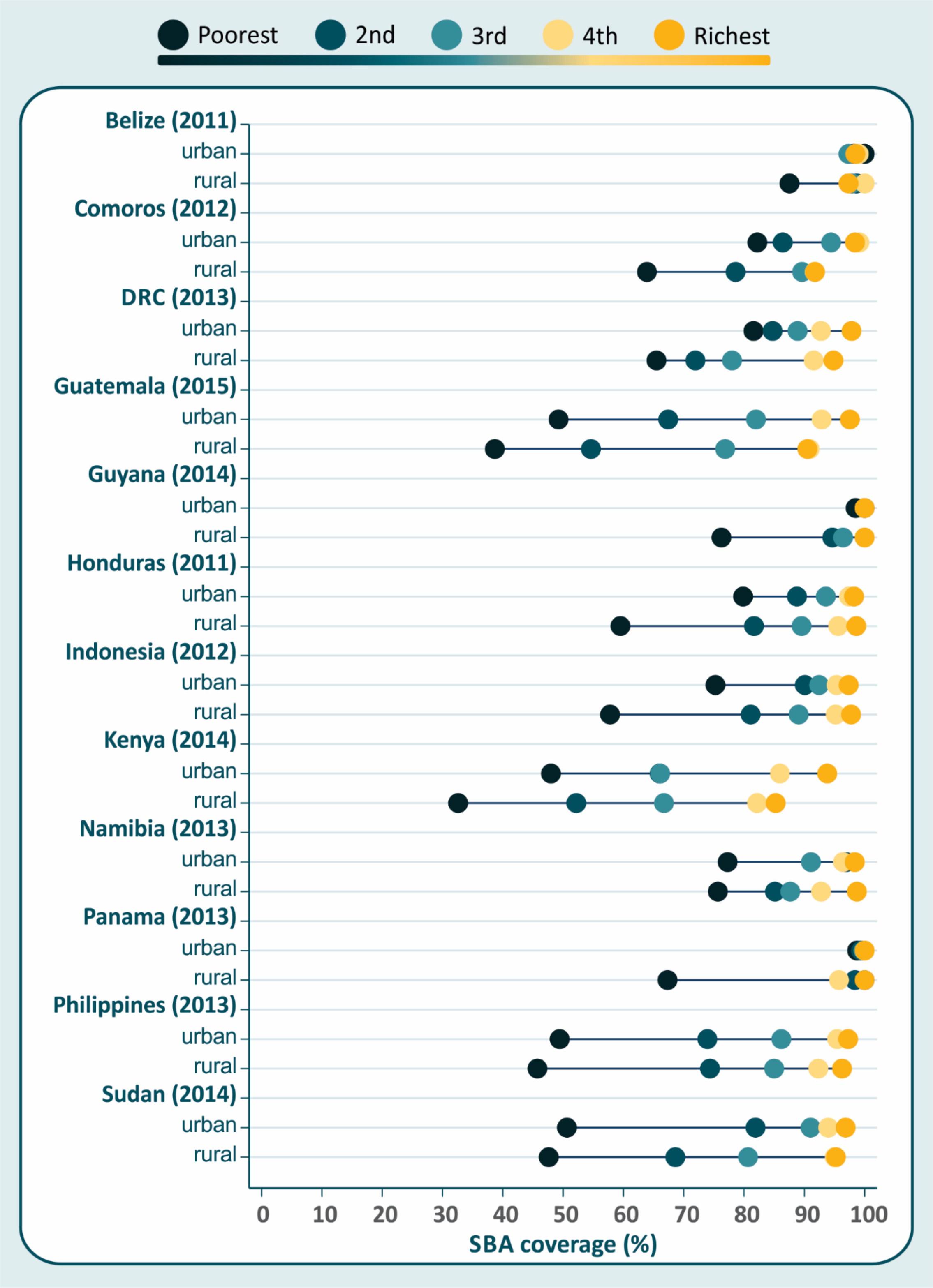

Figure 3 shows a group of countries where urban and rural coverage are similarly high, but with urban inequality advantage. Countries in this group represent 32.4% of all those analyzed. Most countries in this group present a “bottom inequality” pattern rural areas, with the poorest women lagging well behind all other urban and rural groups. The exceptions are Namibia, Philippines and Sudan where coverage seems to be similar for both urban and rural women in the poorest quintile.

Patterns of urban and rural inequalities by region of the world

In Figure 4 (CEE & CIS), countries have reached universal coverage with almost difference between subgroups.

In East Asia (Figure 5), poorest rural women are always behind urban poorest women, and only one country (Thailand) has reached universal coverage.

In Figure 6 (Eastern and Southern Africa) and Figure 10 (West & Central Africa) the situation is a quite bit similar to East Asia, but no countries have reached universal coverage. Almost all rural women were behind the urban women, except for Benin, Namibia and Rwanda where similar coverage are observed in the same poorest quintiles.

Almost all countries in Latin American & Caribbean present similar characteristic in coverage, with all poorest rural women well behind the poorest urban women, and bottom inequality pattern seems to be observed between subgroups, except for the Dominican Republic (Figure 7).

In Middle East & North Africa (Figure 8), countries have reach high coverage except for Sudan where poorest urban and rural women presented similar coverage with wide inequality between subgroups.

In South Asia (Figure 9), no countries have reached universal coverage and similar coverage is observed between urban and poorest rural women, except for Bangladesh. Besides, wide inequality exists in both urban and rural women in all countries.

Supplementary Figure 4: SBA coverage according to the combination of wealth quintile index and urban-rural residence in CEE & CIS.

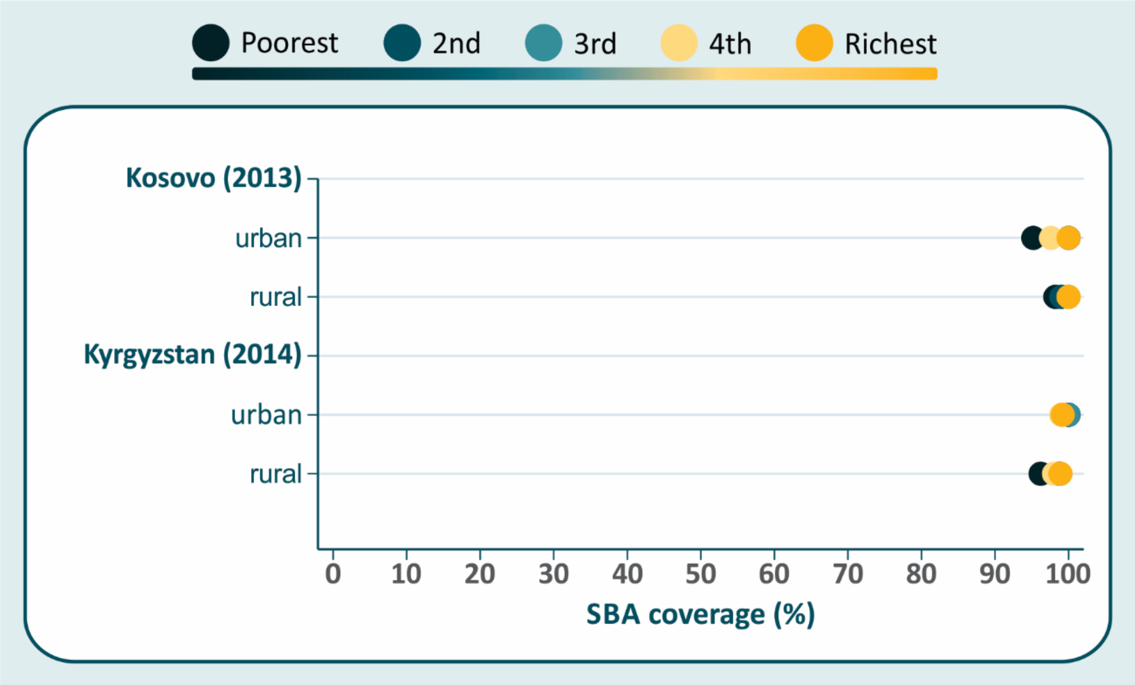

Supplementary Figure 5: SBA coverage according to the combination of wealth quintile index and urban-rural residence in East Asia & Pacific.

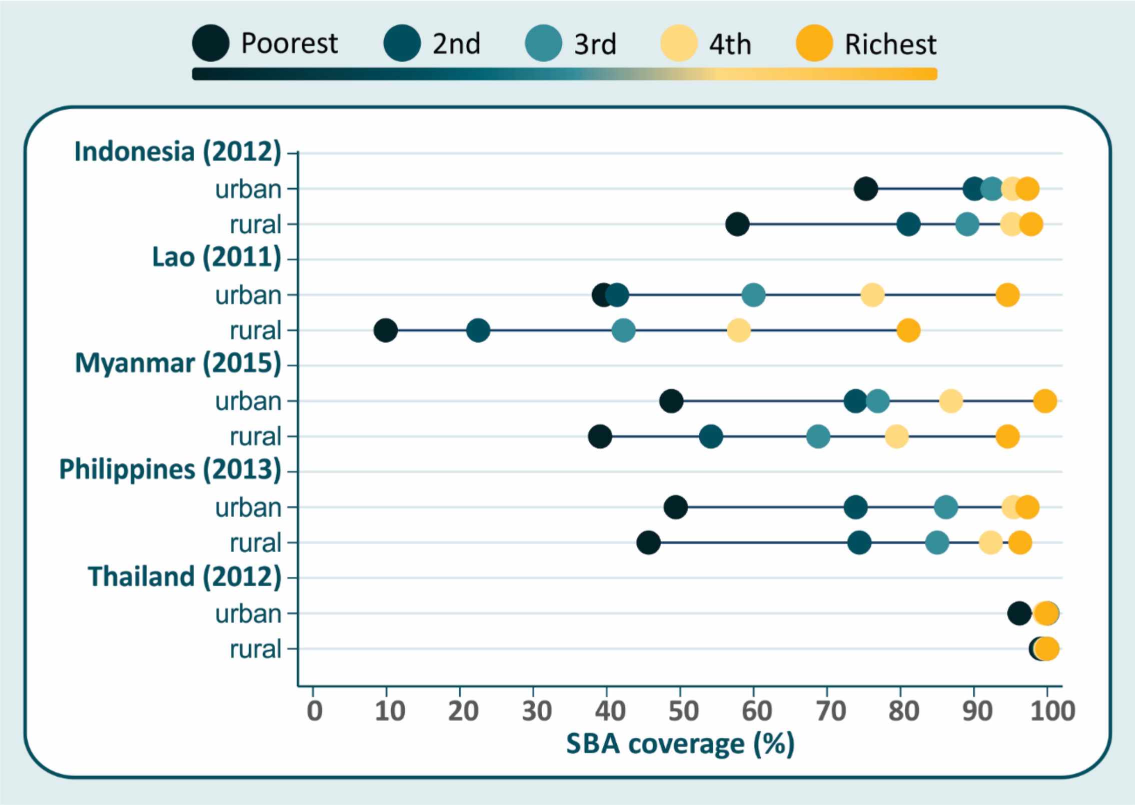

Supplementary Figure 6: SBA coverage according to the combination of wealth quintile index and urban-rural residence in Eastern & Southern Africa.

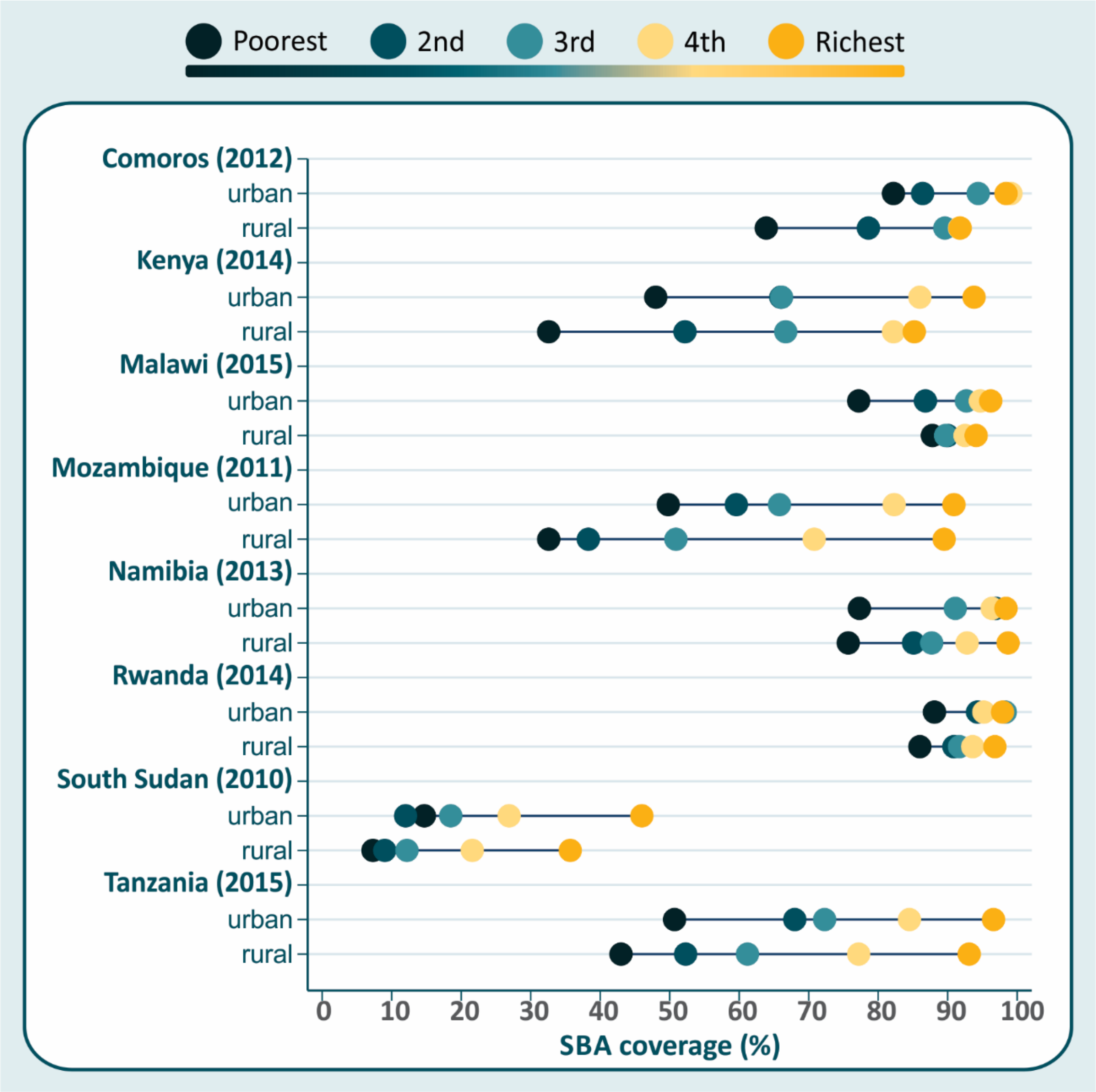

Supplementary Figure 7: SBA coverage according to the combination of wealth quintile index and urban-rural residence in Latin American & Caribbean.

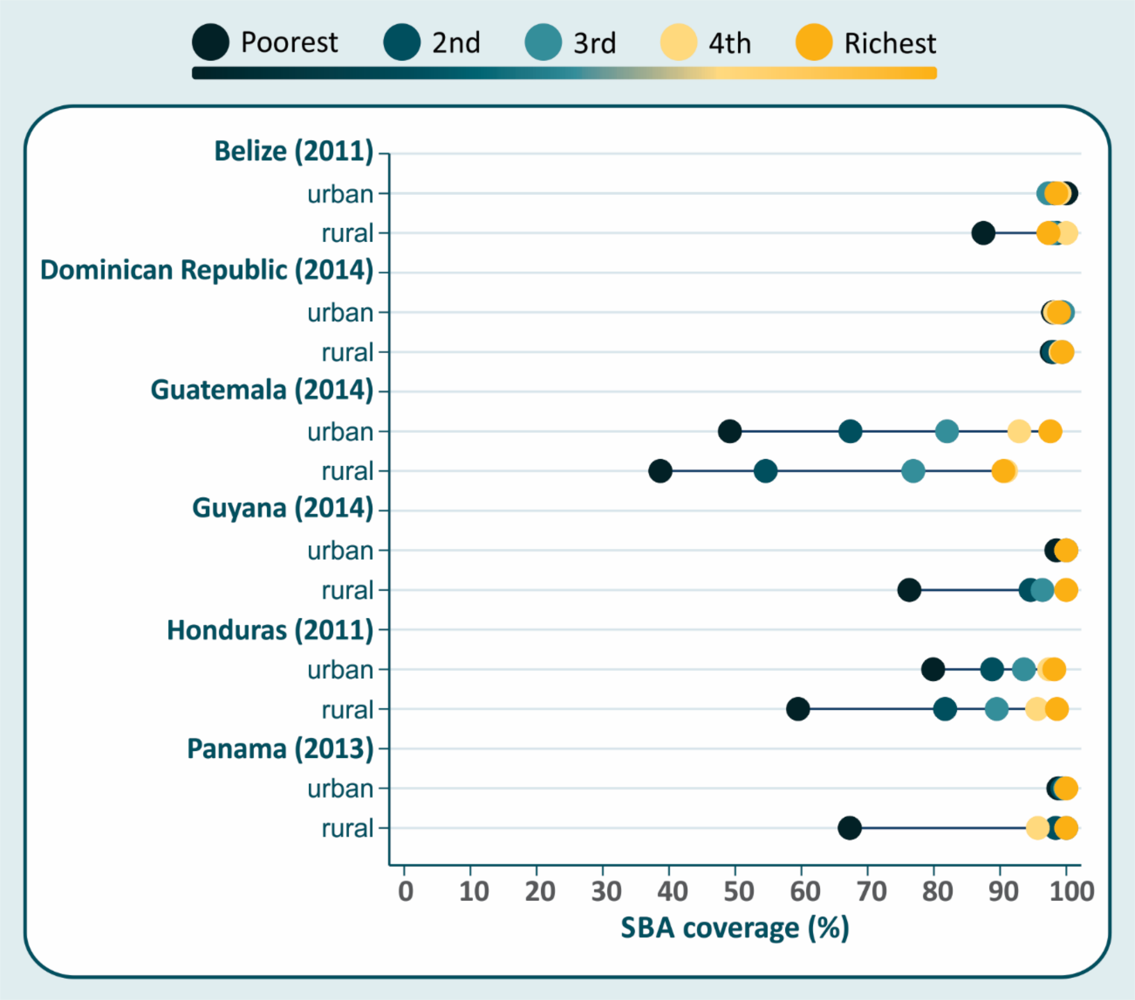

Supplementary Figure 8: SBA coverage according to the combination of wealth quintile index and urban-rural residence in Middle East & North Africa.

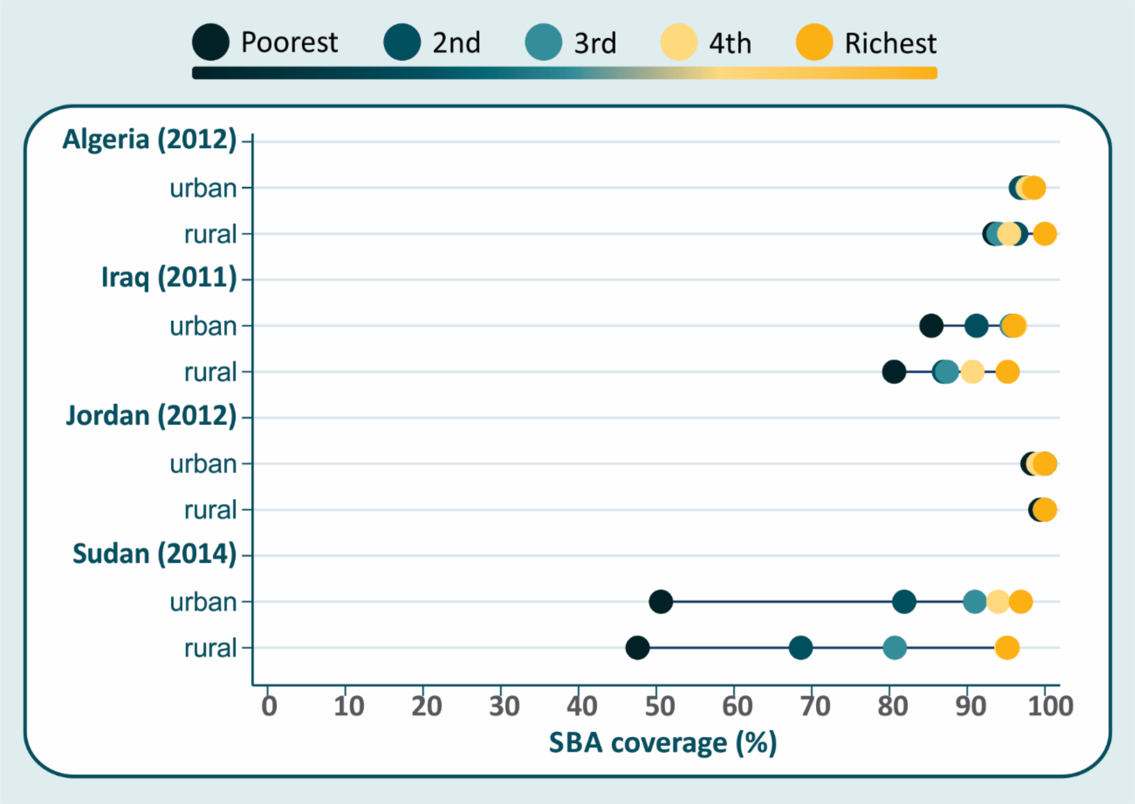

Supplementary Figure 9: SBA coverage according to the combination of wealth quintile index and urban-rural residence in South Asia.

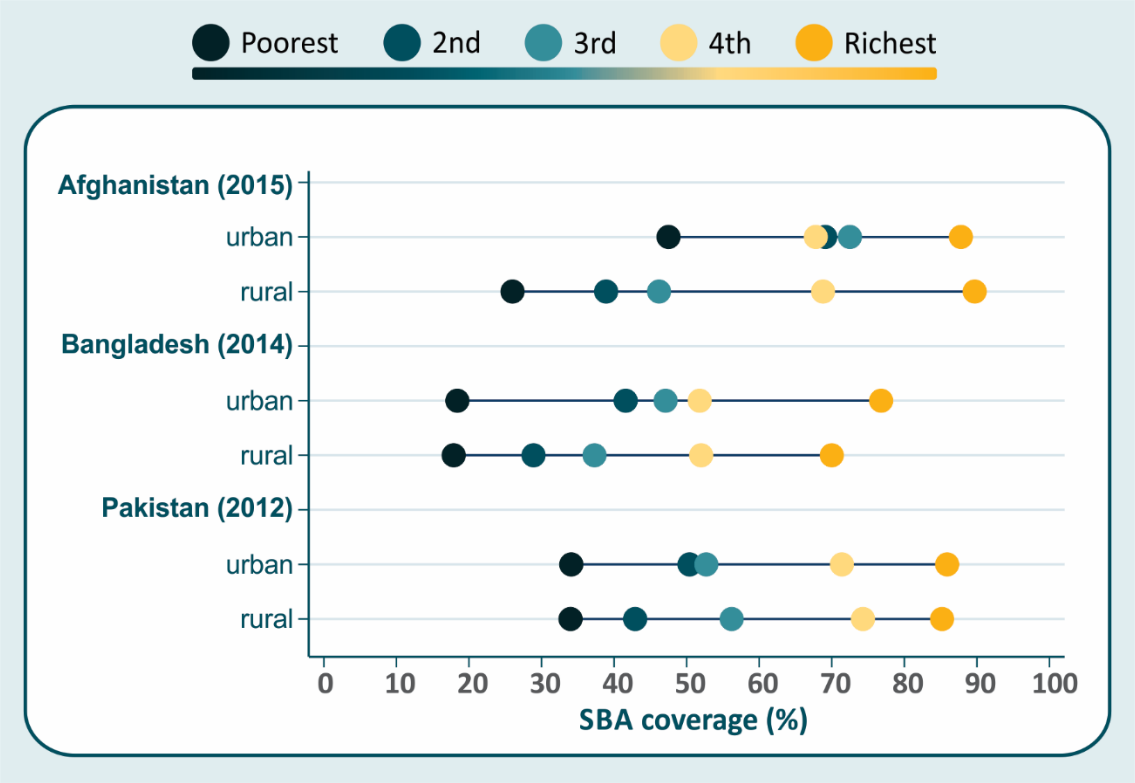

Supplementary Figure 10: SBA coverage according to the combination of wealth quintile index and urban-rural residence in West & Central Africa.

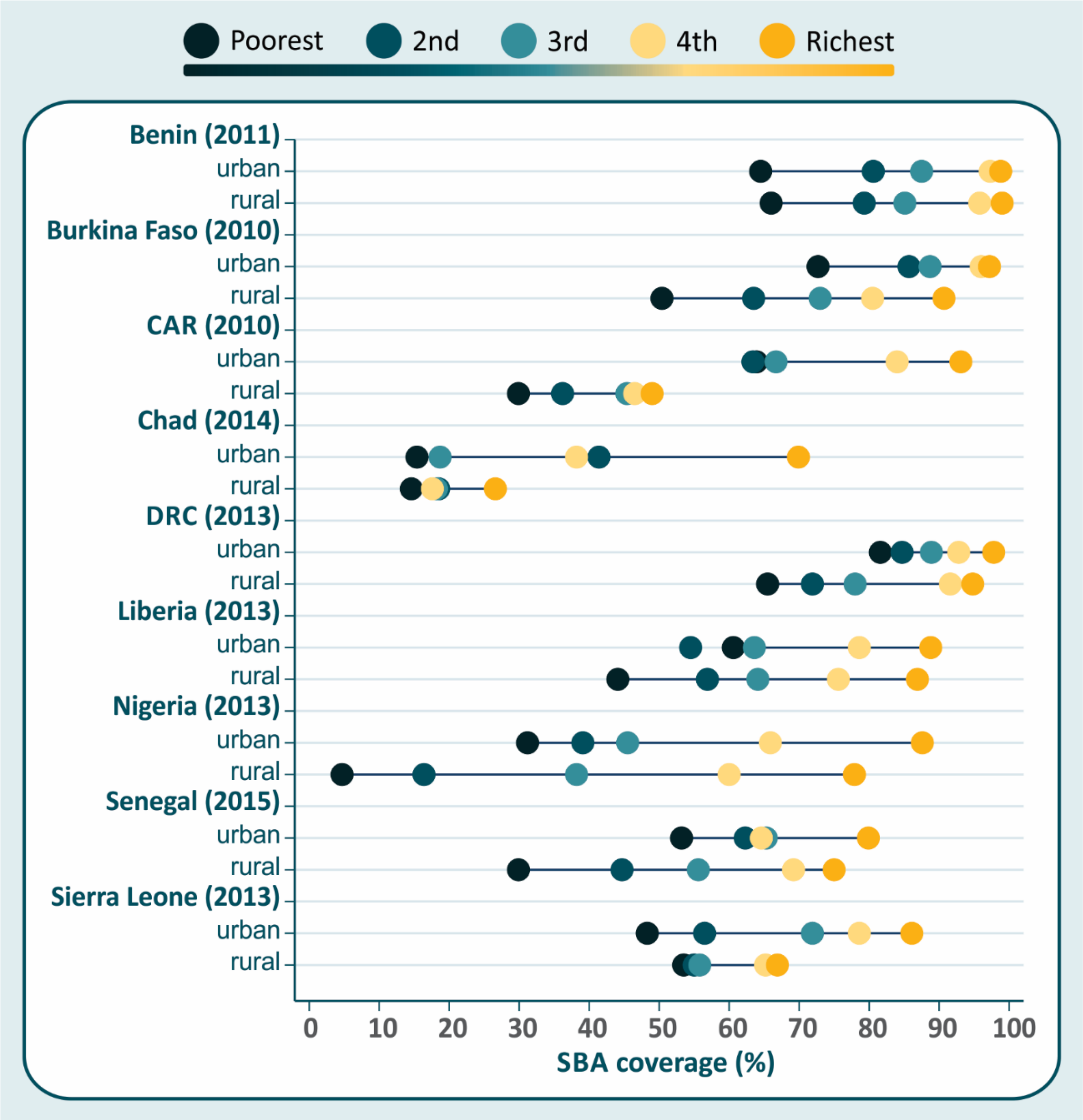

Supplement: Supplementary data [file bmjgh-2018-000898supp004.pdf]
